# DTMUV upregulates DDX17 expression to facilitate viral replication

**DOI:** 10.1186/s13567-025-01639-0

**Published:** 2025-12-16

**Authors:** Yuting Cheng, Wenjing Xie, Qingkang Zhou, Chunyan Ma, Anping Wang, Zhi Wu, Wenfeng Jia, Fan Sun, Fang Zhou, Mixue Wang, Jilin Zhang, Shanyuan Zhu

**Affiliations:** Engineering Technology Research Center for Modern Animal Science and Novel Veterinary Pharmaceutic Development, Jiangsu Key Laboratory of Veterinary Bio-Pharmaceutical High Technology Research, Jiangsu Agri-Animal Husbandry Vocational College, Taizhou, 225300 China

**Keywords:** Duck Tembusu virus, DDX17, viral replication, virus-host interactions, CRISPR/Cas9

## Abstract

Duck Tembusu virus (DTMUV) is a single-stranded positive-sense RNA virus that poses a serious threat to the poultry industry, and its infection mechanism remains incompletely understood. This study aimed to investigate the role of the host protein DDX17 (DEAD-box RNA helicase 17) in DTMUV infection. Utilizing an in vitro infection model combined with CRISPR/Cas9 gene knockout, siRNA interference, and overexpression techniques, we found that DTMUV infection significantly upregulated both mRNA and protein expression levels of DDX17 in host cells. Furthermore, DDX17 facilitated viral replication through its ATP-binding and hydrolysis domain. Co-immunoprecipitation and mass spectrometry analysis confirmed an interaction between DDX17 and the DTMUV C protein. Additionally, phylogenetic analysis revealed that duck DDX17 shares the highest homology (99.8%) with avian orthologs, and tissue distribution assays indicated its highest expression levels in the spleen and liver. This study is the first to reveal the replication-promoting role of DDX17 in avian DTMUV infection and further elucidates its molecular mechanism involving interaction with the viral C protein, providing a theoretical basis for antiviral strategies targeting host factors.

**Supplementary Information:**

The online version contains supplementary material available at 10.1186/s13567-025-01639-0.

## Introduction

Duck Tembusu virus (DTMUV), a member of the *Flavivirus* genus within the *Flaviviridae* family, is a single-stranded positive-sense RNA virus. Its genome is approximately 11 kb in length and encodes three structural proteins and seven non-structural proteins [1, 2]. The earliest known TMUV isolate, MM1775, was obtained from Culex mosquitoes in the Malaysian peninsula in 1955. Subsequent outbreaks occurred in Malaysia and Thailand. DTMUV first emerged in Eastern China in the spring of 2010 and rapidly spread, posing a severe threat to the poultry industry [3, 4]. The virus causes clinical symptoms including sudden drops in egg production, oophoritis, and fatal encephalitis. Following infection, morbidity rates reach 10%–30% in adult ducks and 90%–100% in ducklings, with mortality rates ranging from 5 to 30%. DTMUV has caused annual losses exceeding 5 billion CNY in China’s poultry sector [4, 5]. Studies demonstrate that DTMUV is highly contagious and variable, capable of circulating year-round through diverse transmission routes, including vertical transmission, mosquito bites, direct contact, and aerosol droplets. It exhibits a broad host range, infecting chickens, geese, pigeons, sparrows, and other species. Furthermore, the detection of DTMUV-neutralizing antibodies in the sera of duck farm workers indicates possible cross-species transmission between ducks and humans, suggesting a potential zoonotic risk and a public health concern [6–8]. It is therefore imperative to gain a deeper understanding of its infection mechanisms to develop effective prevention and control strategies. Current research on DTMUV primarily focuses on pathogenicity, epidemiology, vaccine development, and diagnostic methods. However, the molecular mechanisms by which host factors participate in the common processes of viral replication remain poorly understood [9, 10].

Members of the DEAD-box RNA helicase family play crucial roles in RNA metabolism and viral infection [11, 12]. Among them, DDX17 (DEAD-box helicase 17) has obtained significant attention due to its important functions [13]. On one hand, DDX17 participates in host innate immune responses by regulating RNA splicing, miRNA processing, and ribosome biogenesis [14, 15]. On the other hand, it acts as a "double-edged sword" in viral replication, exhibiting diverse functions that can either promote or inhibit infection depending on the pathogen. For instance, DDX17 facilitates human immunodeficiency virus type 1 (HIV-1) replication by binding viral RNA to promote Gag-Pol ribosomal frameshifting, yet it inhibits hepatitis B virus (HBV) proliferation by blocking the packaging of the pregenomic RNA (pgRNA) [16–18]. Furthermore, DDX17 restricts orthobunyavirus replication through its interaction with viral RNA stem-loop structures [19], whereas during H5N1 influenza virus infection, it promotes viral RNA synthesis by interacting with the viral nucleoprotein (NP) [20, 21]. However, the mechanism of DDX17 in avian viruses remains uninvestigated, and its role in infections by members of the *Flavivirus* genus represents uncharted territory.

It was found in our study that DTMUV infection modulates DDX17 expression in host cells. Therefore, this article analyzes the regulation of DTMUV biological activity by the DDX17 protein. This work lays the foundation for subsequent research into the molecular mechanisms of DDX17 and other DEAD-box helicase family members in DTMUV infection. Furthermore, it provides novel insights into pathogen-host interactions and therapeutic strategies against DTMUV infection.

## Materials and methods

### Cell culture and virus infection

DF-1 cells and the DTMUV strain were preserved at the Jiangsu Key Laboratory of Veterinary Bio-pharmaceutical High Technology Research. Cells were cultured in Dulbecco’s Modified Eagle Medium (DMEM) supplemented with 10% fetal bovine serum (FBS) and 1% penicillin/streptomycin (P/S). Cells were maintained at 37 °C in a humidified incubator with 5% CO₂. DF-1 cells in good growth condition were passaged into 12-well plates at a density expected to reach 60–70% confluency the following day. After 24 h, the culture medium was replaced with serum-free DMEM, and DTMUV was inoculated onto the DF-1 cells at the indicated multiplicity of infection (MOI). Following 1 h of viral adsorption, the inoculum was removed, and cells were washed before adding fresh DMEM containing serum. Cells were then returned to the incubator for further culture.

### Antibodies and reagents

KOD One™ PCR Master Mix was purchased from TOYOBO (Shanghai) Biotech Co., Ltd. Restriction endonucleases *Eco*R I and *Xho* I were obtained from New England Biolabs. Gel extraction kits and plasmid extraction kits were acquired from Omega Bio-Tek. The One-Step Fast Cloning Kit (Single Fragment), Alexa Fluor 488-conjugated goat anti-mouse IgG (H + L), and GAPDH monoclonal antibody were sourced from Yeasen Biotechnology (Shanghai) Co., Ltd. DMEM and penicillin/streptomycin were purchased from Gibco. The TransIT-X2® Dynamic Delivery System was purchased from Mirus Bio LLC. FBS was acquired from Invitrogen (USA). The RNA extraction kit and enhanced ECL chemiluminescence detection kit were obtained from Vazyme Biotech Co., Ltd. (Nanjing). Rabbit DDX17 antibody was purchased from HuaAn Biotechnology Co., Ltd. (Hangzhou). Mouse anti-DTMUV C antibody was produced in-house.

### Cell transfection

DF-1 cells in the logarithmic growth phase were harvested and seeded into 12-well plates. When cells reached 80% confluence, transfection was performed according to the manufacturer’s instructions for the X-tremeGENE HP DNA Transfection Reagent. For each transfection, 100 μL of Opti-MEM medium and 1 μL of TransIT-X2 transfection reagent were added to a 1.5 mL microcentrifuge tube. Plasmid DNA (1 μg) was added to one tube, while an equivalent amount of control plasmid was added to the other tube. The solutions in both tubes were mixed thoroughly and incubated for 15 min at room temperature to allow complex formation. The resulting complexes were then added dropwise to the respective wells of the 12-well plate. Each well was supplemented with fresh, pre-warmed medium containing 10% fetal bovine serum (without antibiotics) to a final volume of 1 mL. Following transfection for the designated time period, cells were harvested for subsequent analyses.

### siRNA knockdown

The specific small interfering RNA (siRNA) targeting DDX17 (siDDX17) used in this study was obtained from GenePharma (Shanghai, China). DF-1 cells in good growth condition were passaged into 12-well plates at a density expected to reach 80–90% confluency the following day. Using Transfection Buffer as the diluent, siRNA (siRNA gene sequences are listed in Table 1) and PepMute™ siRNA Transfection Reagent were diluted, mixed gently, and incubated at room temperature for 20 min. The mixture was then added dropwise to the wells of the 12-well plate. Cells were subsequently returned to the incubator for further culture.

**Table 1:** **DDX17 siRNA gene sequence**

Name	Sequence (5'–3')	Sequence (3'–5')
siDDX17#1	GGACCAGAAUUUCACAGAATT	UUCUGUGAAAUUCUGGUCCTT
siDDX17#2	GCUCUCAGUGGUCGUGAUATT	UAUCACGACCACUGAGAGCTT
siDDX17#3	GCUGAGUGCCAACCACAAUTT	AUUGUGGUUGGCACUCAGCTT
siDDX17#4	GCCAGCUAUGUGUAUCCAUTT	AUGGAUACACAUAGCUGGCTT

### CRISPR/Cas9

A DDX17-knockout (KO) DF-1 cell line was established using CRISPR/Cas9 technology. The plasmids pGL3-U6-sgRNA-PGK-puromycin and pST1374-NLS-flag-linker-Cas9 were employed to construct the DDX17-KO cell line. Guide RNA (gRNA) sequences targeting chicken DDX17 were designed using the Zhang Lab online tool [22] (Table 2). Successfully constructed gRNAs were transfected into DF-1 cells. At 48 h post-transfection, cells were co-cultured with puromycin (2 μg/mL) and blasticidin S (BSD) (20 μg/mL). After 8 d of selection, a subset of cells was harvested, and target gene expression was assessed by western blotting. DF-1 cells exhibiting DDX17 downregulation were subjected to single-cell cloning via limiting dilution in 96-well plates. Endogenous DDX17 expression levels were verified by western blotting.

**Table 2:** **DDX17-gRNA gene sequence**

Name	Sequence (5'–3')	Sequence (3'–5')
gDDX17#1	ACCGGTAACCCGGGGGAACGCTTA	AAACTAAGCGTTCCCCCGGGTTAC
gDDX17#2	ACCGTTGGAGGAAGAGGACCTCCA	AAACTGGAGGTCCTCTTCCTCCAA
gDDX17#3	ACCGCCCGGGTTACCAAATTTCTT	AAAC AAGAAATTTGGTAACCCGGG
gDDX17#4	ACCGCCAAAGAAATTTGGTAACCC	AAACGGGTTACCAAATTTCTTTGG

### Infection of ducklings

We confirm that all animal experiments were conducted in accordance with the Guidelines for the Care and Use of Laboratory Animals established by Jiangsu Agri-animal Husbandry Vocational College. The ethical approval for this study was obtained from the Institutional Animal Care and Use Committee of Jiangsu Agri-animal Husbandry Vocational College. Twenty healthy 7-day-old ducklings, confirmed to be free from DTMUV and its antibodies by qRT-PCR and ELISA, were used. The ducklings were randomly allocated into two groups (*n* = 10 per group). Ducklings in the DTMUV group were intramuscularly inoculated with DTMUV (0.2 mL per duckling). Ducklings in the control group were intramuscularly inoculated with 0.2 mL of sterile phosphate-buffered saline (PBS) as a negative control. At 5 days post-infection (dpi), all surviving ducklings were euthanized. Various tissues were collected and subjected to qRT-PCR analysis.

### Quantitative real-time PCR (qRT-PCR)

To measure the RNA levels of specific genes, total intracellular RNA was extracted from cells using TRNzol Universal Reagent (TIANGEN). First-strand cDNA was synthesized from the extracted RNA using the RevertAid First Strand cDNA Synthesis Kit (Thermo Fisher Scientific). cDNA quantification was performed by qRT-PCR using Bestar® SybrGreen qPCR Master Mix (DBI® Bioscience). The presented data represent the relative abundance of target RNAs normalized to GAPDH. Nucleic acid gel stain (Super™ GelRed, #S-2001) was obtained from Everbright® Inc (USA). All qRT-PCR experiments were conducted on an ABI 7500 Real-Time PCR System (Applied Biosystems). Primers used for qRT-PCR detection are listed in Table 3.

**Table 3:** **Primers used for qRT-PCR detection**

Gene	FP (5'–3')	RP (5'–3')
*DTMUV*	GGTTTTGTGCCACTAGCGTG	GCCTTGCTTTCTTTCCACGG
*DDX17*	GGATGGCCCAATTTGCTTGG	GATCTGGGGACCTTTGGGTG
*GAPDH*	ACTTCAACGGTGACAGCCAT	ACCATCAAGTCCACCACACG

### Co-immunoprecipitation and immunoblotting

Treated and untreated cells were collected and lysed on ice for 30 min using pre-chilled NHG lysis buffer (1% Triton X-100, 10% glycerol, 50 mM HEPES, pH 7.2, 150 mM NaCl) supplemented with protease and phosphatase inhibitors. Cell debris was removed by centrifuging the extracts at 12 000 rpm for 15 min. Total protein concentration was determined using the Pierce™ BCA Protein Assay Kit (Invitrogen). Cell lysates were incubated with specific primary antibodies at room temperature for 1 h, followed by overnight incubation with Dynabeads® Protein G magnetic beads at 4 °C. Beads were washed five times with wash buffer (1% Triton X-100, 10% glycerol, 50 mM HEPES, pH 7.2, 400 mM NaCl) to remove non-specifically bound proteins. Equal amounts of protein were separated by electrophoresis on 10% SDS–polyacrylamide gels and transferred onto nitrocellulose (NC) membranes. Membranes were blocked with 5% non-fat dry milk for 2 h at room temperature, then probed with primary antibodies overnight at 4 °C and corresponding horseradish peroxidase (HRP)-conjugated secondary antibodies for 2 h at room temperature. Target protein bands were finally detected by chemiluminescence using the WesternBright™ ECL kit (Advansta).

### Immunoprecipitation coupled with mass spectrometry (IP-MS)

DF-1 cells infected with DTMUV at MOI = 1 for 48 h were lysed on ice for 30 min using pre-chilled NHG lysis buffer supplemented with protease and phosphatase inhibitors. Cell debris was removed by centrifugation at 12 000 rpm for 15 min. The supernatant was incubated with anti-DTMUV C antibody at room temperature for 1 h, followed by overnight incubation with Dynabeads® Protein G magnetic beads at 4 °C. Beads were washed five times with wash buffer to remove non-specifically bound proteins. Proteins were eluted by adding 30 μL of elution buffer (0.15 M glycine, pH 2.5–3.1) and incubating at room temperature for 10 min. Beads were magnetically separated, and the supernatant was transferred to a new microcentrifuge tube. The eluate was immediately neutralized by spiking with neutralizing buffer (0.1 M NaOH) at one-tenth of the total volume, adjusting the pH to physiological range. Processed samples were stored at −80 °C for subsequent functional analysis. LC–MS/MS detection and bioinformatic analysis were performed by BGI Genomics Co., Ltd. (Shenzhen, China).

### Confocal microscopy analysis

Cells were washed three times with PBS, fixed with pre-cooled 4% paraformaldehyde at room temperature for 15 min, permeabilized with 0.1% Triton X-100 for 10 min, and blocked with 5% bovine serum albumin (BSA) for 30 min. Primary antibodies were incubated with cells overnight at 4 °C. Subsequently, cells were incubated with Alexa Fluor-conjugated secondary antibodies for 2 h at room temperature, followed by nuclear counterstaining with 4′,6-diamidino-2-phenylindole (DAPI). Fluorescence images were captured using a confocal laser-scanning microscope (Leica TCS SP2). EGFP and Alexa Fluor 488 were excited at 488 nm, Cy3 at 561 nm, and DAPI at 408 nm, using sequential scanning mode to avoid crosstalk.

### Statistical analysis

Graphical representations and statistical analysis were performed using Adobe Photoshop CS6, Image J, and GraphPad Prism 6 software. Data are presented as mean ± standard deviation (SD) from at least three independent biological replicates. Statistical significance was determined using unpaired two-tailed Student’s *t*-tests. Significance thresholds were defined as follows: *P* < 0.05 (*), *P* < 0.01 (**), and *P* < 0.001 (***).

## Results

### Phylogenetic analysis of DDX17 protein

**Figure 1:**
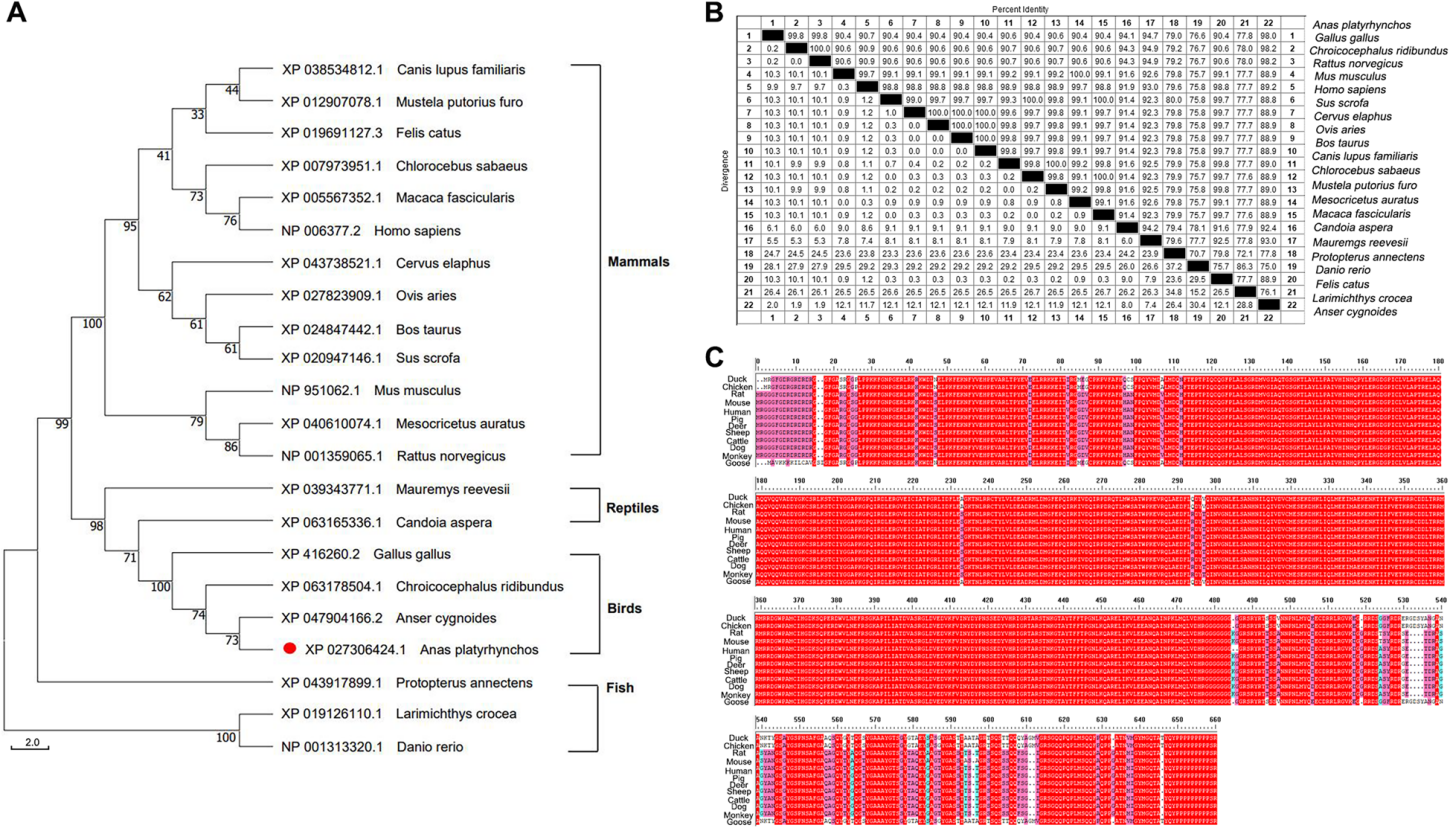
**Phylogenetic analysis of duck DDX17**. **A** Phylogenetic tree of DDX17 orthologs. The tree illustrates evolutionary relationships among DDX17 sequences from duck (Anas platyrhynchos) and diverse vertebrates, constructed using the Neighbor-Joining method in MEGA-X with 1000 bootstrap replicates. Scale bar: 0.050 substitutions per site. **B** Sequence similarity of DDX17 between duck and representative species. **C** Multiple sequence alignment of DDX17 amino acid residues across species.

Amino acid sequences from multiple species were aligned using MEGA-X software. A phylogenetic tree depicting the evolutionary relationships of DDX17 genes across diverse species was constructed using the Neighbor-Joining (NJ) method (Figure 1A). Species included in the analysis encompassed reptiles, fish, mammals, and birds. Phylogenetic analysis revealed the degree of sequence identity between duck DDX17 and orthologs from other species. Specifically, duck DDX17 exhibited 99.8% sequence identity with chicken (*Gallus gallus*), 99.8% with black-headed gull (*Chroicocephalus ridibundus*), and 79% with West African lungfish (*Protopterus annectens*). The highest sequence identity was observed between duck and both chicken and black-headed gull (Figures 1B, C), all belonging to the avian class.

### Tissue-specific expression profile of DDX17 in vivo

**Figure 2:**
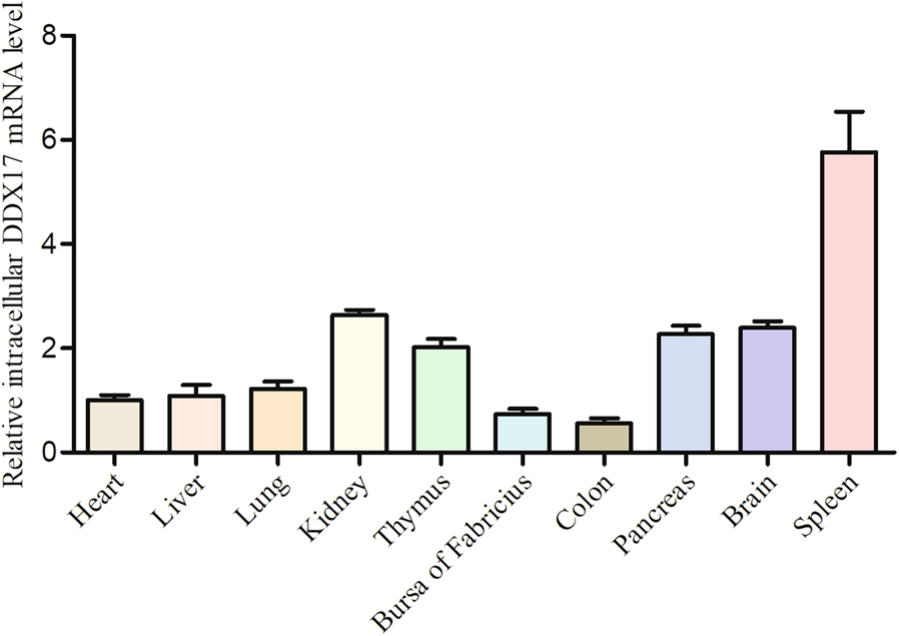
**Tissue distribution profile of duck DDX17**. Relative DDX17 mRNA expression across tissues from healthy Cherry Valley ducks was quantified by qRT-PCR. Expression levels were normalized to the endogenous GAPDH reference gene and calibrated to heart tissue (set as 1.0). Relative gene expression was calculated using the 2^−ΔΔct^ method. Data represent mean values from three biological replicates.

To comprehensively characterize the tissue distribution of duck DDX17 in healthy Cherry Valley ducks, key tissues—including heart, liver, lung, kidney, thymus, bursa of Fabricius, colon, pancreas, brain, and spleen—were collected for total RNA extraction. The relative expression levels of DDX17 across these tissues were quantified using qRT-PCR. Expression data were normalized to cardiac tissue as the reference. Results demonstrated ubiquitous DDX17 expression with the highest transcript levels detected in the spleen, followed by kidney, brain, pancreas, and thymus. Comparatively lower expression was observed in the bursa of Fabricius and colon (Figure 2).

### Upregulation of DDX17 during DTMUV infection

**Figure 3:**
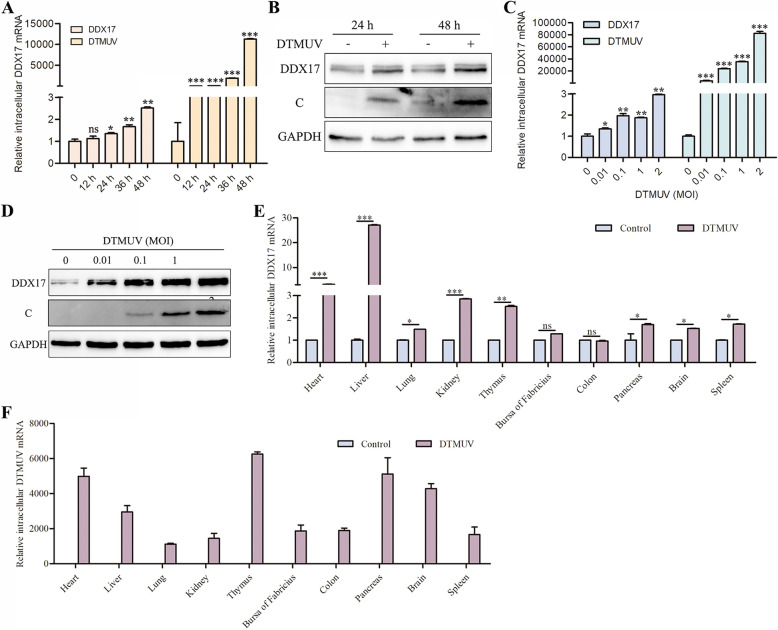
**DTMUV infection induces DDX17 expression**. **A**, **B** Time-course analysis of DDX17 expression in DF-1 cells infected with DTMUV (MOI = 0.1). mRNA and protein levels were assessed by qRT-PCR and western blotting at the indicated time points. Representative blots show DDX17 protein with β-actin loading control. **C**, **D** Dose-responsive DDX17 expression in DF-1 cells infected with increasing MOI of DTMUV (0, 0.01, 0.1, 1, 2) for 48 h. **E**, **F** Relative DDX17 or DTMUV mRNA quantification in tissues from healthy vs. DTMUV-infected ducks. Data normalized to heart tissue. Error bars represent SD of three biological replicates. Statistical significance: ns, not significant; *, *P* < 0.05; **,* P* < 0.01; ***, *P* < 0.001 (unpaired Student’s *t*-test).

To evaluate the potential role of DDX17 in DTMUV infection, we examined the impact of viral challenge on endogenous DDX17 expression. First, we assessed time-dependent changes by inoculating DF-1 cells with DTMUV strain (MOI = 0.1) and collecting samples at 12, 24, 36, and 48 h post-infection (hpi) for qRT-PCR analysis, with parallel western blotting at 24 and 48 hpi. Results demonstrated significant increases in both DDX17 mRNA expression (Figure 3A) and protein abundance (Figure 3B) proportional to infection duration. Second, we evaluated dose-dependent effects by infecting DF-1 cells with increasing DTMUV titers (MOI = 0, 0.01, 0.1, 1, and 2). Samples collected at 48 hpi revealed stepwise upregulation of DDX17 mRNA (Figure 3C) and protein levels (Figure 3D) with higher viral inocula. Furthermore, we determined tissue-specific DDX17 transcription in healthy and DTMUV-infected ducks. qRT-PCR analysis of heart, liver, spleen, kidney, brain, pancreas, lung, and thymus showed ubiquitous DDX17 upregulation in infected tissues. Notably, the liver exhibited the most pronounced induction (27.1-fold increase vs. healthy controls; Figures 3E and F). Collectively, these findings demonstrate that DTMUV infection significantly induces DDX17 expression both in vitro and in vivo.

### DDX17 facilitates DTMUV replication in DF-1 cells

**Figure 4:**
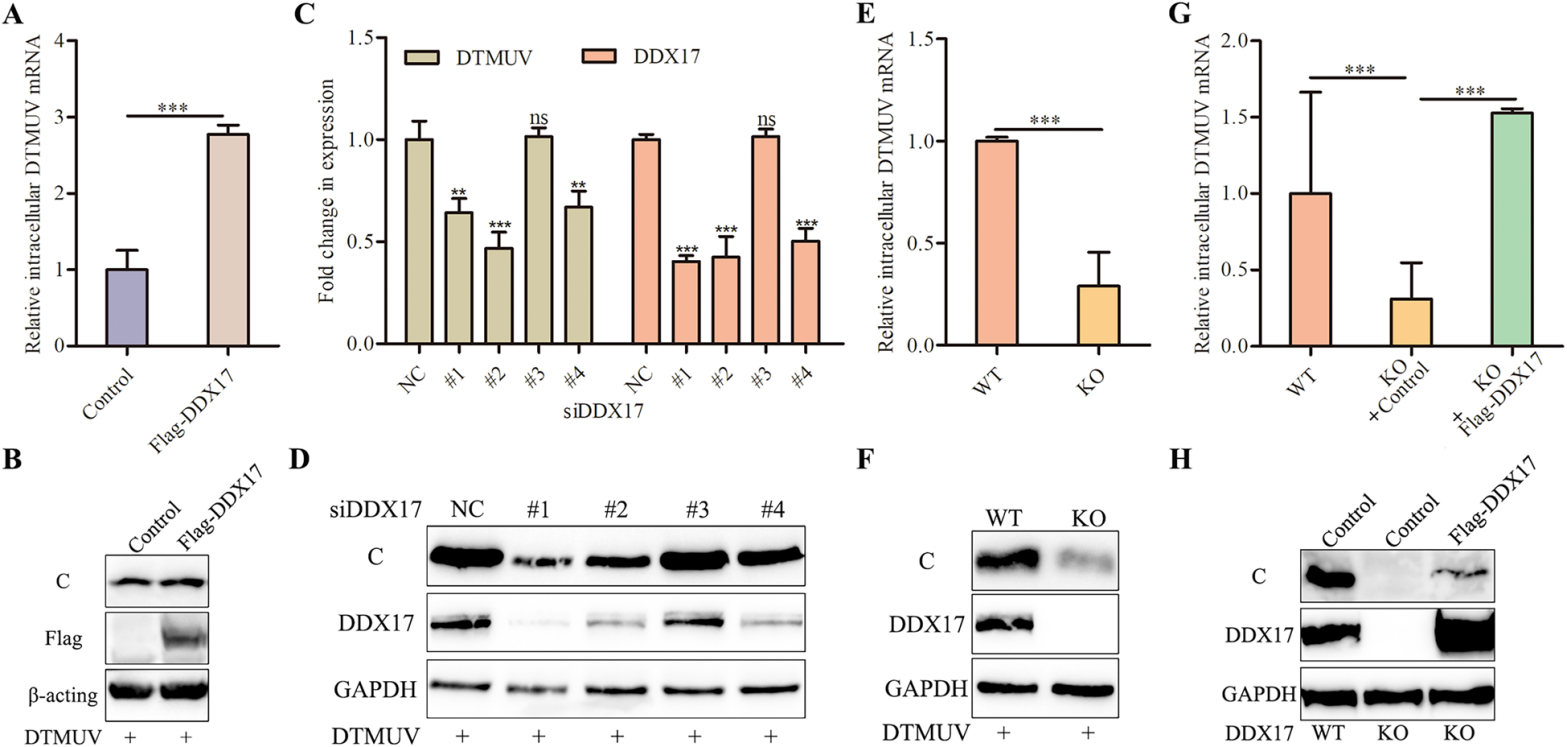
**DDX17 promotes DTMUV replication**. **A**, **B** Enhanced DTMUV replication upon DDX17 overexpression. DF-1 cells transfected with empty vector or Flag-DDX17 plasmids were infected with DTMUV (MOI = 0.1) at 24 h post-transfection. Viral mRNA levels and C protein expression were assessed by qRT-PCR and western blotting. **C**, **D** Suppressed DTMUV replication following DDX17 knockdown. Cells transfected with control siRNA (siNC) or DDX17-targeting siRNAs were infected with DTMUV (MOI = 0.1) at 24 h post-transfection. **E**, **F** Impaired viral replication in DDX17- KO cells. WT and KO DF-1 cells were infected with DTMUV (MOI = 0.1). **G**, **H** Rescue of viral replication by DDX17 reconstitution. DDX17-KO cells were reconstituted with Flag-DDX17 prior to DTMUV infection (MOI = 0.1). Data represent mean ± SD of three biological replicates. Statistical significance: ns, not significant; *, *P* < 0.05; **,* P* < 0.01; ***, *P* < 0.001 (unpaired Student’s *t*-test).

To determine the impact of DDX17 on DTMUV replication, DF-1 cells were transfected with plasmids encoding Flag-tagged DDX17 prior to viral infection. Compared to the empty vector control, DDX17-overexpressing cells exhibited significantly higher levels of viral mRNA and C protein (Figures 4A, B), indicating that DDX17 overexpression enhances DTMUV replication. To investigate the role of endogenous DDX17, four siRNAs targeting distinct regions of DDX17 mRNA were designed. qRT-PCR and Western blotting confirmed that siDDX17-#1, #2, and #4 substantially reduced endogenous DDX17 levels. Concurrently, these knockdowns significantly diminished DTMUV mRNA and C protein expression post-infection (Figures 4C, D), demonstrating that DDX17 suppression inhibits viral replication. For further validation, a DDX17- KO DF-1 cell line was established using CRISPR/Cas9. Consistent with siRNA results, KO cells showed reduced DTMUV mRNA and C protein levels versus WT controls (Figures 4E, F). Crucially, reintroduction of Flag-DDX17 into KO cells rescued viral replication (Figures 4G, H). Collectively, these findings establish DDX17 as a proviral host factor essential for efficient DTMUV replication in avian cells.

### DDX17 facilitates DTMUV replication via its ATP-Binding domain

**Figure 5:**
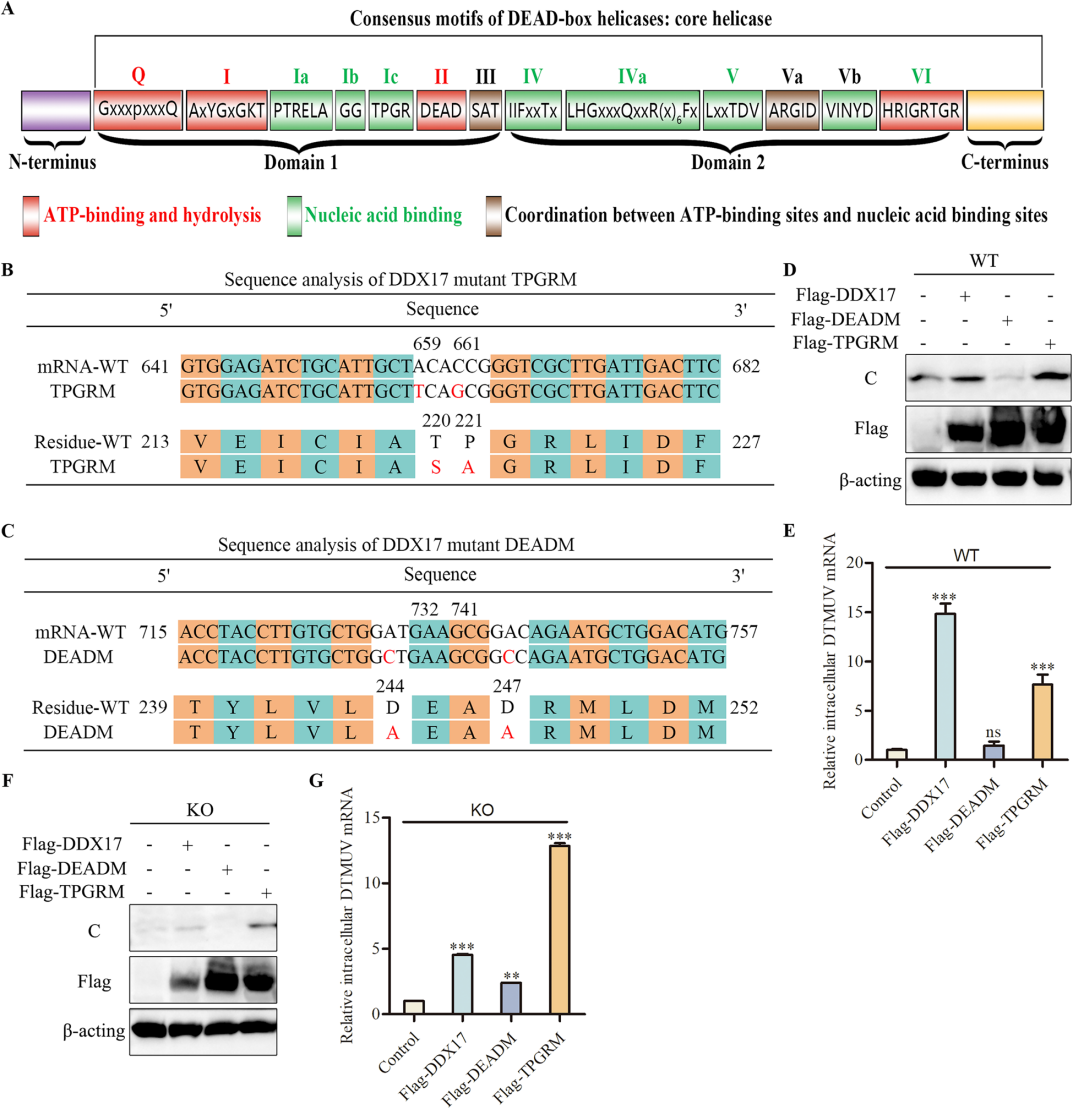
**DDX17 facilitates DTMUV replication via its ATP-binding domain.** **A** Domain architecture of DExH/D-box helicase core. Conserved motifs involved in ATP binding/hydrolysis (red), nucleic acid binding (blue), or inter-domain coordination (red/blue stripes) are shown. The DEAD-box helicase consensus sequence (x = any amino acid) is indicated for each motif. **B**, **C** Sequence alignment analysis of DDX17 mutants. Site-directed mutagenesis introduced nucleotide substitutions A659T/C661G (TPGRM: T220S/P221A) and A732C/A741C (DEADM: D244A/D247A). **D**–**G** Requirement of ATP-binding domain for DDX17-mediated viral enhancement. WT (**D**, **E**) and DDX17-KO (**F**, **G**) cells were transfected with vector, Flag-DDX17, Flag-DEADM, or Flag-TPGRM plasmids. At 24 h post-transfection, cells were infected with DTMUV (MOI = 1) for 48 h. Viral mRNA and C protein levels were quantified by qRT-PCR and western blotting. Data: Mean ± SD of three biological replicates. Statistical significance: ns, not significant; *, *P* < 0.05; **,* P* < 0.01; ***, *P* < 0.001 (unpaired Student’s *t*-test).

The DDX17 helicase core comprises conserved ATP-binding and RNA-binding domains (Figure 5A). Within these, motif Ic (TPGR) in the RNA-binding domain and motif II (DEAD) in the ATP-binding domain exhibit the highest conservation [23]. To identify the minimal functional region mediating DDX17-DTMUV interaction, we generated point mutations in these motifs: TPGRM (TPGR → SAGR) and DEADM (DEAD → AEAA) (Figures 5B, C). WT and DDX17-KO DF-1 cells were transfected with plasmids expressing DDX17, TPGRM, DEADM, or empty vector, followed by DTMUV infection. In both WT (Figures 5D, E) and KO cells (Figures 5F, G), overexpression of DDX17 or TPGRM significantly enhanced viral mRNA and C protein levels compared to vector controls. Conversely, DEADM failed to increase viral replication despite equivalent expression. These results demonstrate that DDX17 potentiates DTMUV replication specifically through its ATP-binding domain (DEAD motif).

### DDX17 interacts with the DTMUV C protein

**Figure 6:**
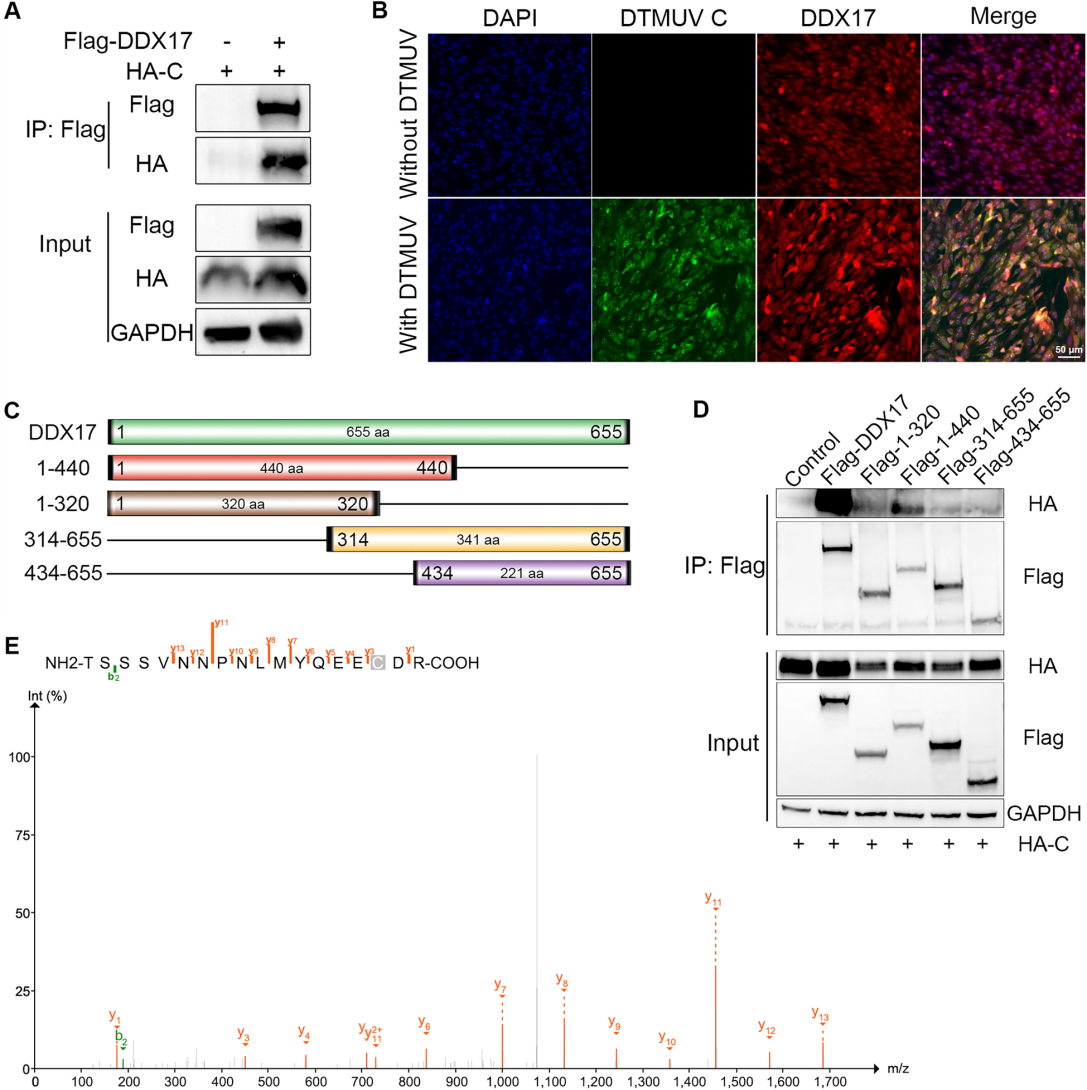
**DDX17 interacts with the DTMUV C protein. A** Co-immunoprecipitation (Co-IP) of DDX17 and C protein in HEK 293 T cells. Cells co-transfected with Flag-DDX17 and HA-C plasmids were subjected to IP with anti-Flag antibody, followed by immunoblotting (IB) with anti-HA and anti-Flag antibodies. **B** Subcellular co-localization of DDX17 and C protein in DTMUV-infected DF-1 cells. Cells fixed with 4% PFA were stained with anti-DDX17 (red), anti-C (green), and DAPI (blue). Yellow: Co-localization signals. Scale bar: 10 μm (Z-stack projection). **C** Schematic of full-length and truncated DDX17 constructs. **D** Mapping the DDX17-C interaction domain. HEK 293 T cells co-transfected with HA-C and truncated Flag-DDX17 plasmids were processed for Flag-Co-IP. **E** IP-MS validating DDX17-derived peptides in C protein complexes. In DTMUV-infected DF-1 cells, IP using anti C antibody followed by MS identified DDX17-derived peptides, confirming physical interaction between DDX17 and the viral capsid protein.

To elucidate how DDX17 facilitates DTMUV replication, co-immunoprecipitation (Co-IP) assays were performed to identify interacting viral partners. DTMUV C protein was detected in DDX17 immunoprecipitates (Figure 6A). Confocal microscopy confirmed cytoplasmic co-localization of DDX17 and C protein during infection (Figure 6B). To map the interaction domain, truncated DDX17 constructs were generated (Figure 6C). The N-terminal fragment (1–440) retained binding capability to C protein, albeit at reduced efficiency compared to full-length DDX17. Fragments 1–320, 341–655, and 434–655 completely lost binding activity (Figure 6D). Complementary IP-MS analysis of DTMUV-infected DF-1 lysates immunoprecipitated with anti-C antibody identified DDX17-derived peptides (Figure 6E). Collectively, these results demonstrate interaction between DDX17 and the DTMUV C protein.

## Discussion

DEAD-box helicase DDX17 is implicated in cancer, viral pathogenesis, and ontogenesis [14, 15, 24]. Mounting evidence indicates that DDX helicases critically regulate viral replication: DDX1 exhibits context-dependent restriction/promotion of viruses [25–28]; DDX3 suppresses type I IFN production while facilitating replication of diverse viruses, emerging as a key host target [29, 30]; DDX5 functions as a proviral cofactor during RNA virus infections [31]. In contrast, DDX17’s role in viral replication remains incompletely characterized. DDX17 plays multifaceted roles in RNA metabolism, including RNA splicing, translation, degradation, and ribosome biogenesis [14, 15]. DDX17 mRNA is alternatively translated into two isoforms, p72 and p82. Given their nearly identical properties, most studies have not differentiated their functions [32]. Functionally, DDX17 displays virus-specific modulation: It serves as an intrinsic host restriction factor against HBV by blocking pgRNA encapsidation [16], yet promotes H5N1 influenza viral RNA synthesis in human cells [20, 21]. Notably, it exhibits dual regulatory roles in HIV infection – enhancing replication through RNA metabolism modulation [18] while acting as a cofactor for antiviral protein ZAP [33]. This functional divergence likely stems from differential exploitation of host factors across viral lifecycles. Our study establishes DDX17 as a novel pro-viral factor facilitating DTMUV replication, expanding its repertoire within the virus-host interaction landscape.

As a member of the *Flavivirus* genus, DTMUV relies heavily on host RNA helicases for viral RNA replication. DDX17 may enhance replication efficiency by facilitating viral RNA processing or modulating host immune recognition. Our study revealed that DDX17 undergoes no significant nucleocytoplasmic translocation during DTMUV infection despite marked upregulation proportional to infection duration and viral load. Notably, this contrasts with observations in other viral systems, providing new perspectives on DDX17’s functional dynamics. Prior studies report DDX17 translocation from nucleus to cytoplasm during Hantavirus infection—a strategy for evading host immune surveillance [34]. However, in DTMUV-infected DF-1 cells, DDX17 remains predominantly nuclear without observable redistribution. This fundamental divergence suggests distinct virus-specific mechanisms: While DDX17 exploits nucleocytoplasmic shuttling to support Hantavirus immune evasion, its nuclear retention during DTMUV infection implies alternative proviral mechanisms, potentially through direct interactions with viral components in the nuclear compartment.

The helicase core of DDX17 comprises conserved ATP-binding/hydrolysis and RNA-binding domains. Within these, motif Ic (TPGR) in the RNA-binding domain and motif II (DEAD) in the ATPase domain exhibit highest conservation [23, 35, 36]. While hepatic DDX17 demonstrates anti-HBV activity dependent on RNA-binding functionality [16], our functional assays establish that the ATPase domain (DEAD motif) is essential for DDX17’s pro-viral role in DTMUV replication, whereas mutation of the RNA-binding domain (TPGR motif) exerted no significant effect on viral replication efficiency. This mechanistic divergence suggests that DDX17 exerts virus-specific functions through distinct molecular interfaces: its RNA-binding domain enables host defense against cytoplasmic HBV via pgRNA interference, whereas its ATPase activity is co-opted by nucleus-replicating DTMUV. This represents a novel adaptation strategy within the Flaviviridae family.

The pro-viral function of DDX17 in DTMUV contrasts sharply with its restriction of HBV, underscoring that viral exploitation of host helicases is dictated by genome biology and subcellular niches. Whereas HBV—a nuclear DNA virus—is inhibited by DDX17’s RNA-binding domain, cytoplasmic RNA viruses (DTMUV, influenza) co-opt its ATPase activity to enhance replication. This mechanistic divergence positions DDX17 as a context-dependent arbiter of viral success, offering new targets for pathogen-specific antivirals.

In summary, this study reveals the distinct proviral role of DDX17 in DTMUV infection. Contrasted with its functions in other viral systems, our findings provide novel insights into the functional versatility of DDX17 during viral pathogenesis. These results establish a foundation for developing antiviral strategies targeting host DDX17. Future research should delineate: Structural determinants governing DDX17-capsid protein interactions. Collectively, this work advances our understanding of flavivirus-host interplay and provides a theoretical framework for host-directed antiviral therapeutics.

## Supplementary Information

**Additional file** **1**. **Full uncropped gels and blots image(s).**

## Data Availability

The data that supported the findings of this study are available from the authors upon reasonable request.

## Abbreviations

DTMUV: Duck Tembusu virus
DDX17: DEAD-box RNA helicase 17
HBV: hepatitis B virus
pgRNA: pregenomic RNA
HIV-1: human immunodeficiency virus type 1
NP: nucleoprotein
DMEM: Dulbecco’s modified eagle medium
FBS: fetal bovine serum
P/S: penicillin/streptomycin
MOI: multiplicity of infection
BSD: blasticidin
gRNA: guide RNA
KO: knockout
qRT-PCR: quantitative real-time PCR
NC: nitrocellulose
HRP: horseradish peroxidas
IP-MS: immunoprecipitation coupled with mass spectrometry
BSA: bovine serum albumin
hpi: hours post-infection
Co-IP: co-immunoprecipitation
