# Supplementary material for: DTMUV upregulates DDX17 expression to facilitate viral replication
Source: Vet Res. 2025 Dec 16;56:230. doi: 10.1186/s13567-025-01639-0 (PMC12709809; doi:10.1186/s13567-025-01639-0)
Supplement: Supplementary file 1 — Additional file 1. Full uncropped gels and blots image(s). [file 13567_2025_1639_MOESM1_ESM.pptx]

## Slide 1
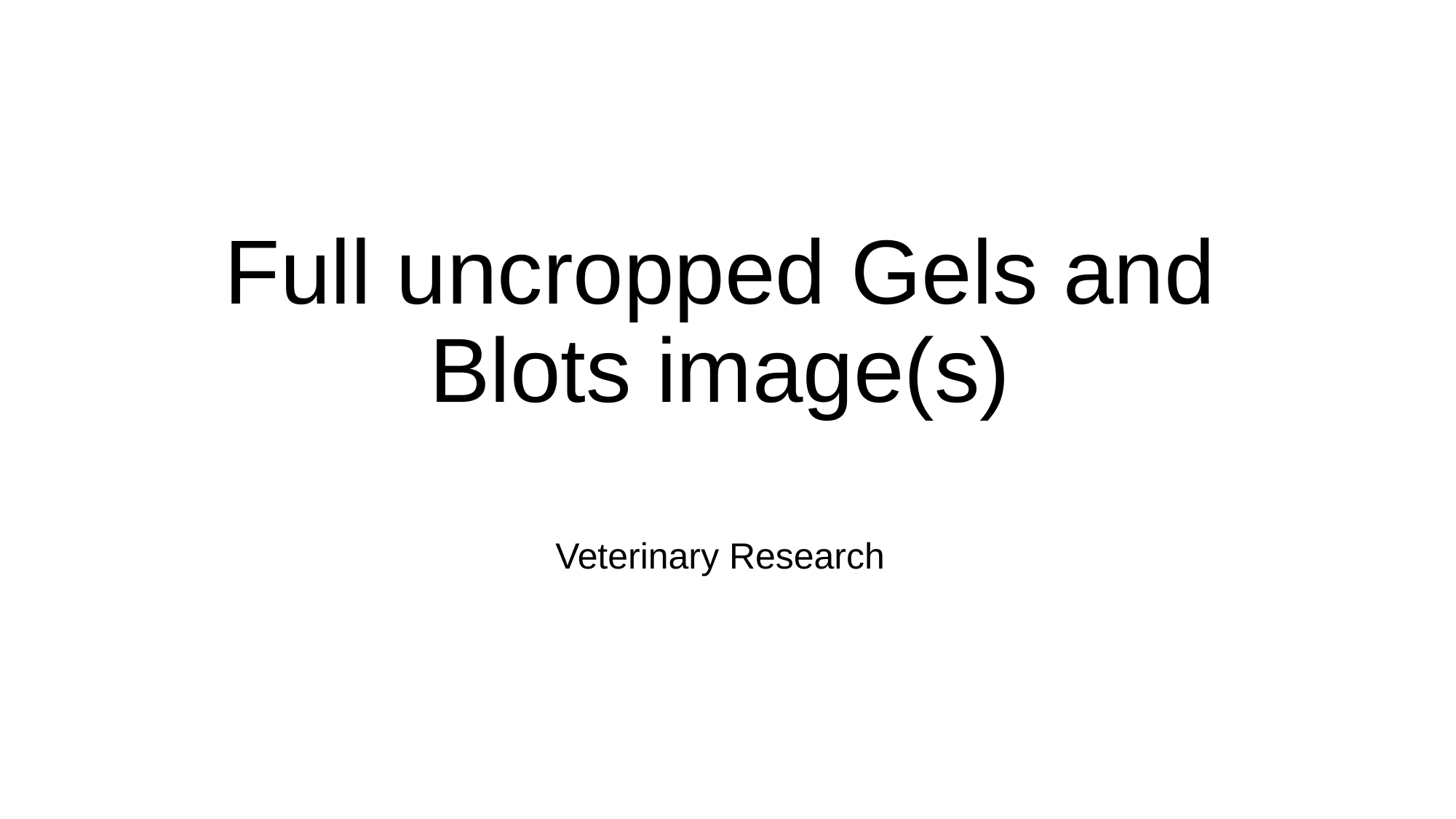

# Full uncropped Gels and Blots image(s)
Veterinary Research

## Slide 2
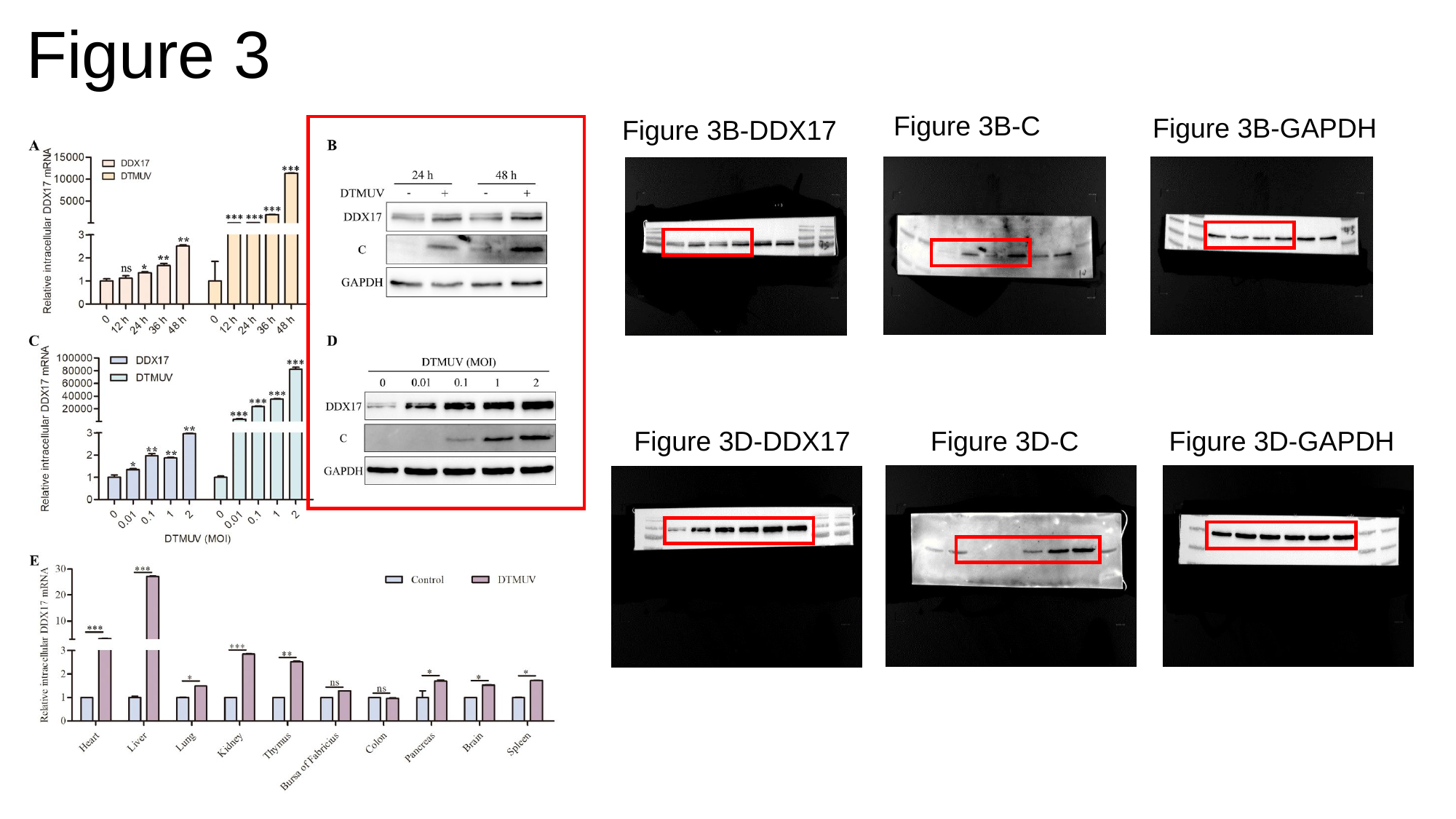

# Figure 3
Figure 3B-C
Figure 3B-GAPDH
Figure 3B-DDX17
Figure 3D-DDX17
Figure 3D-GAPDH
Figure 3D-C

## Slide 3
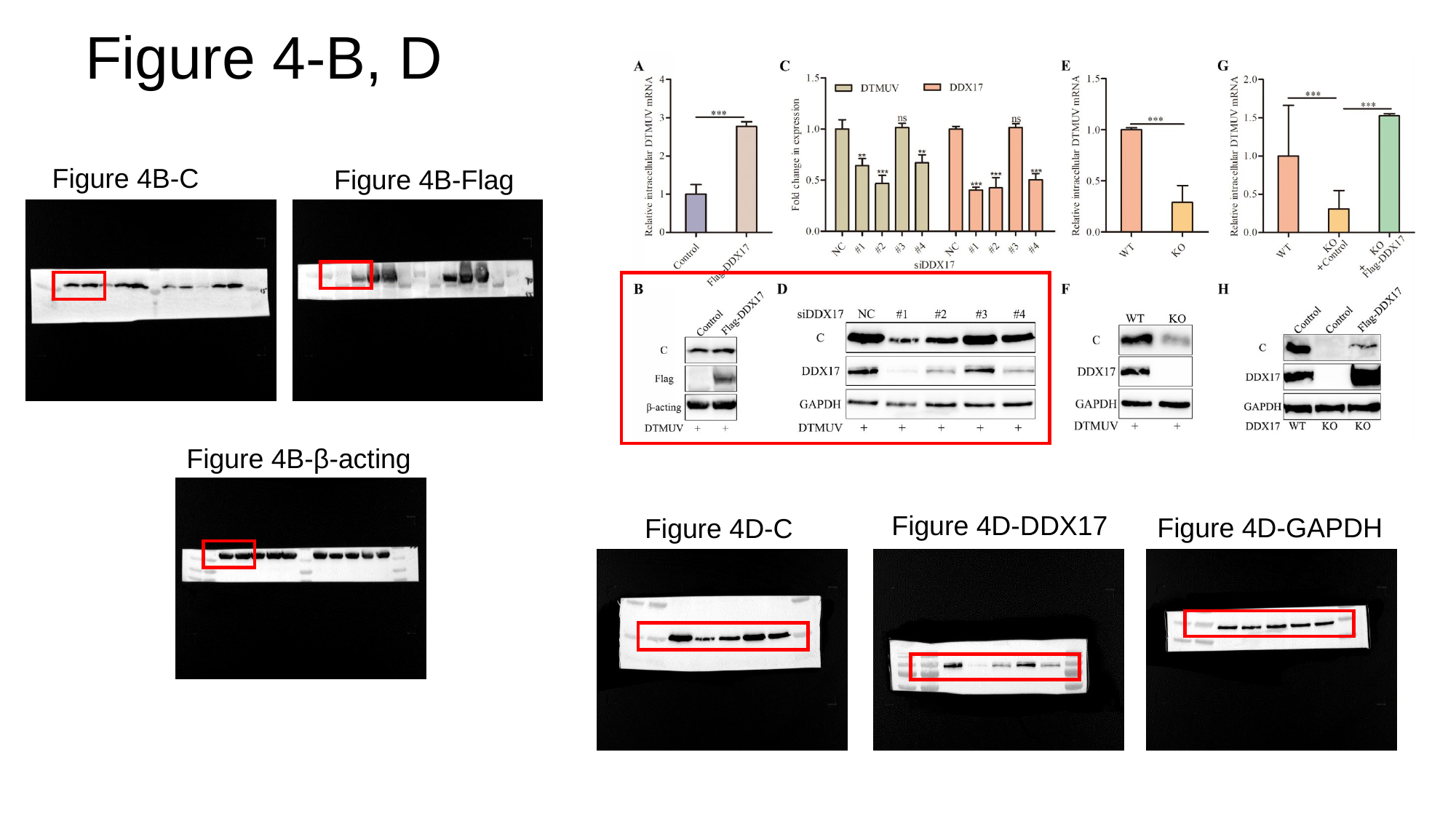

# Figure 4-B, D
Figure 4B-C
Figure 4B-Flag
Figure 4B-β-acting
Figure 4D-DDX17
Figure 4D-GAPDH
Figure 4D-C

## Slide 4
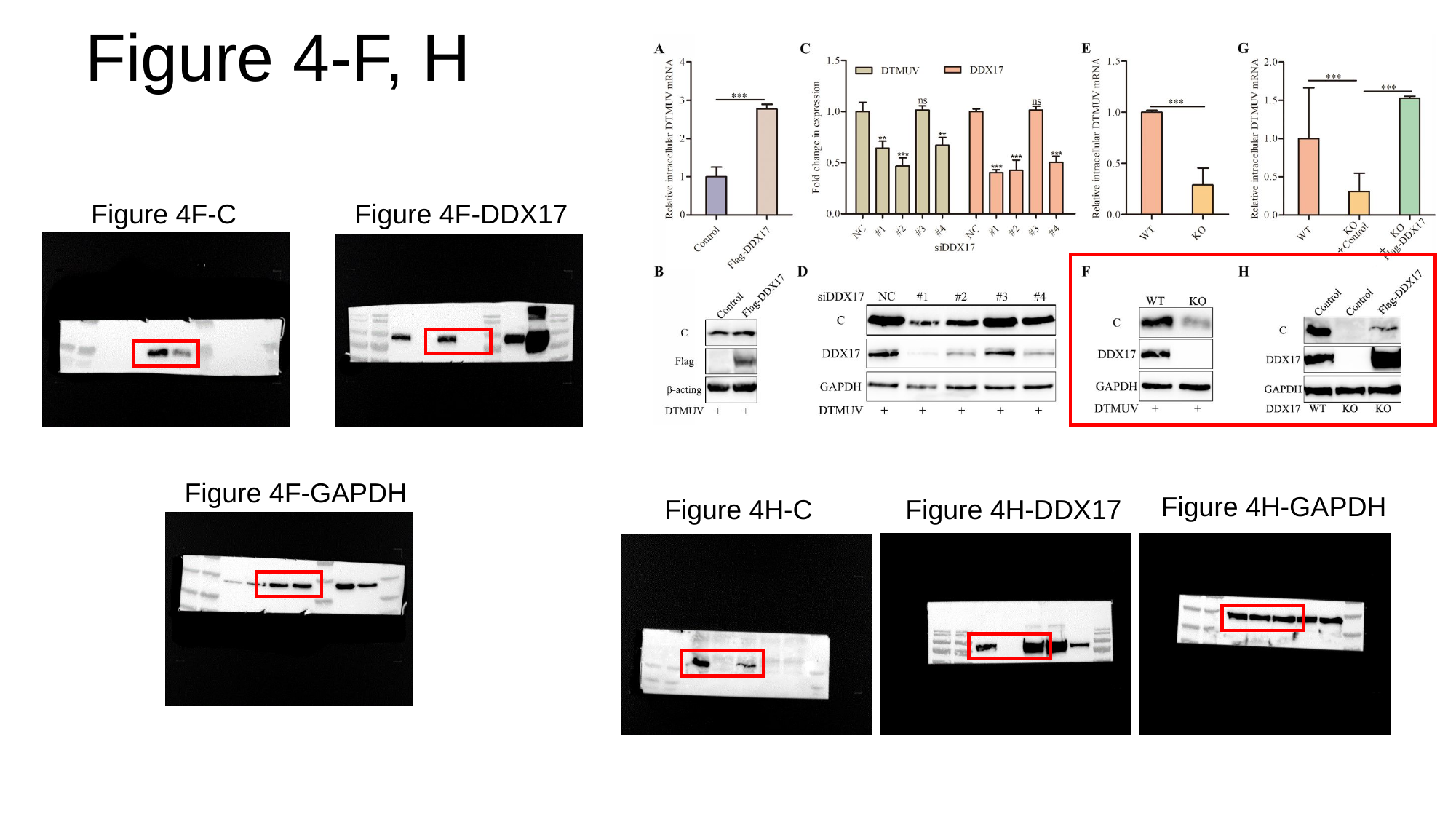

# Figure 4-F, H
Figure 4F-C
Figure 4F-DDX17
Figure 4F-GAPDH
Figure 4H-GAPDH
Figure 4H-C
Figure 4H-DDX17

## Slide 5
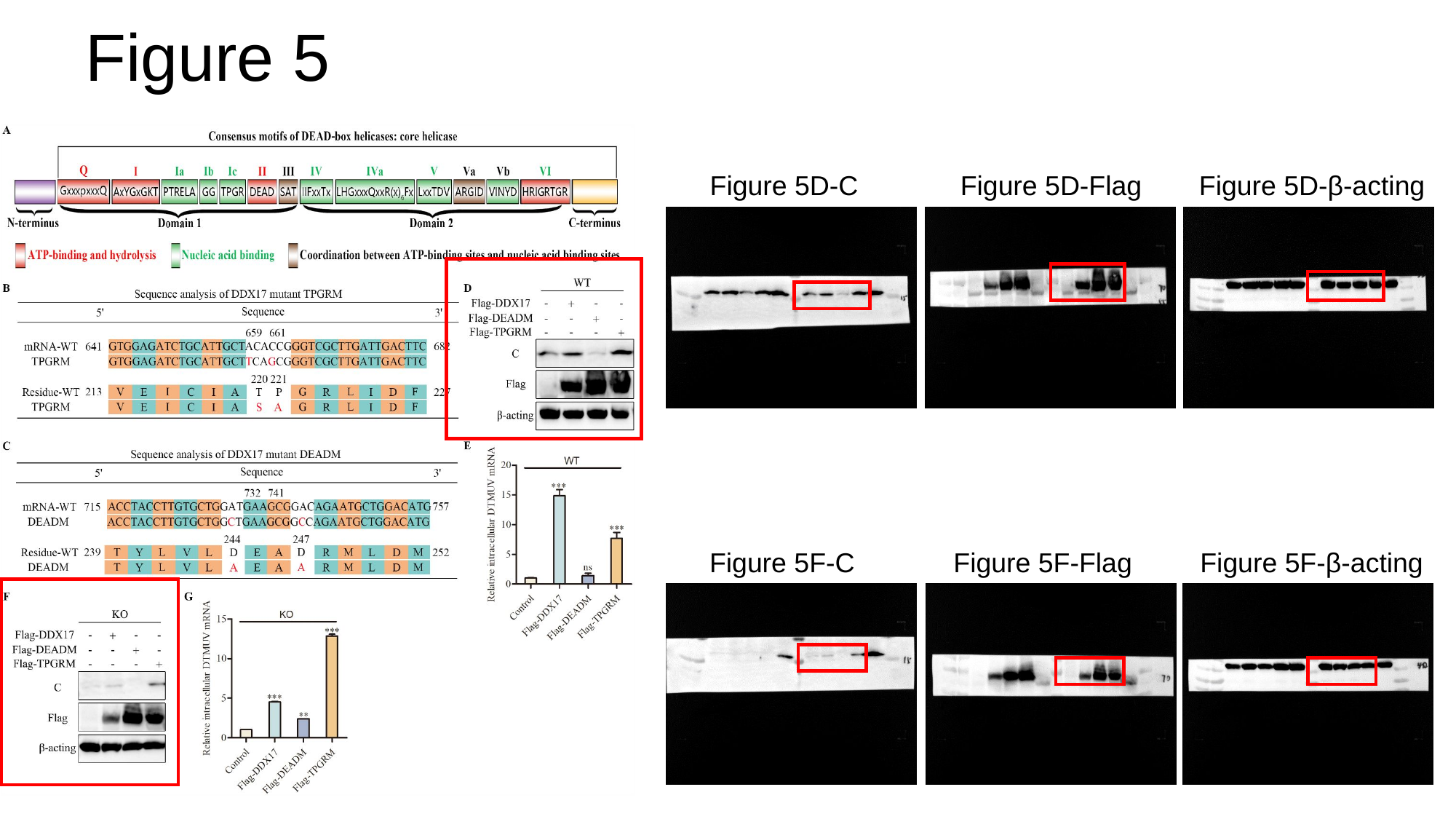

# Figure 5
Figure 5D-Flag
Figure 5D-β-acting
Figure 5D-C
Figure 5F-Flag
Figure 5F-β-acting
Figure 5F-C

## Slide 6
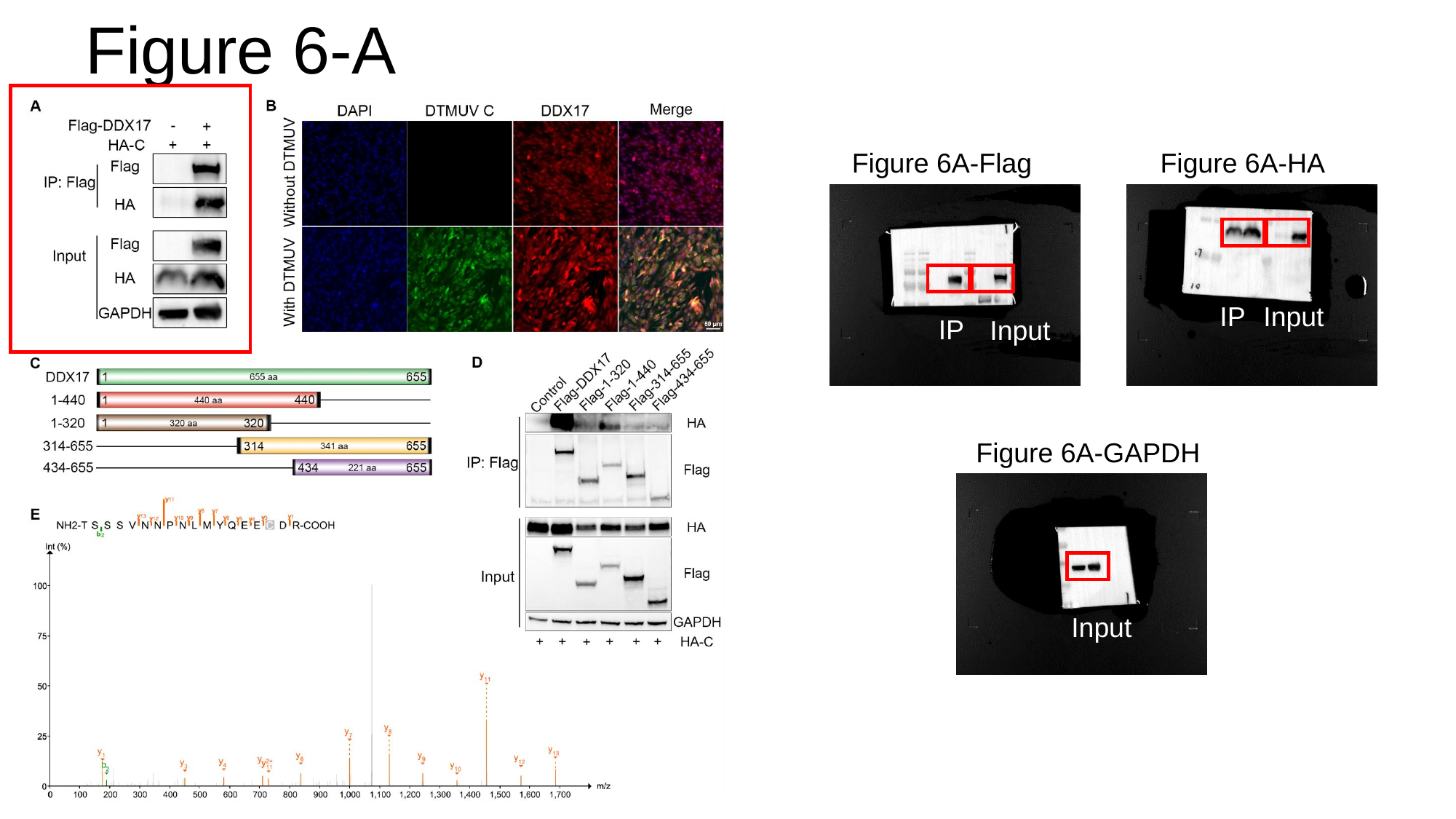

# Figure 6-A
Figure 6A-HA
IP
Input
Figure 6A-Flag
IP
Input
Figure 6A-GAPDH
Input

## Slide 7
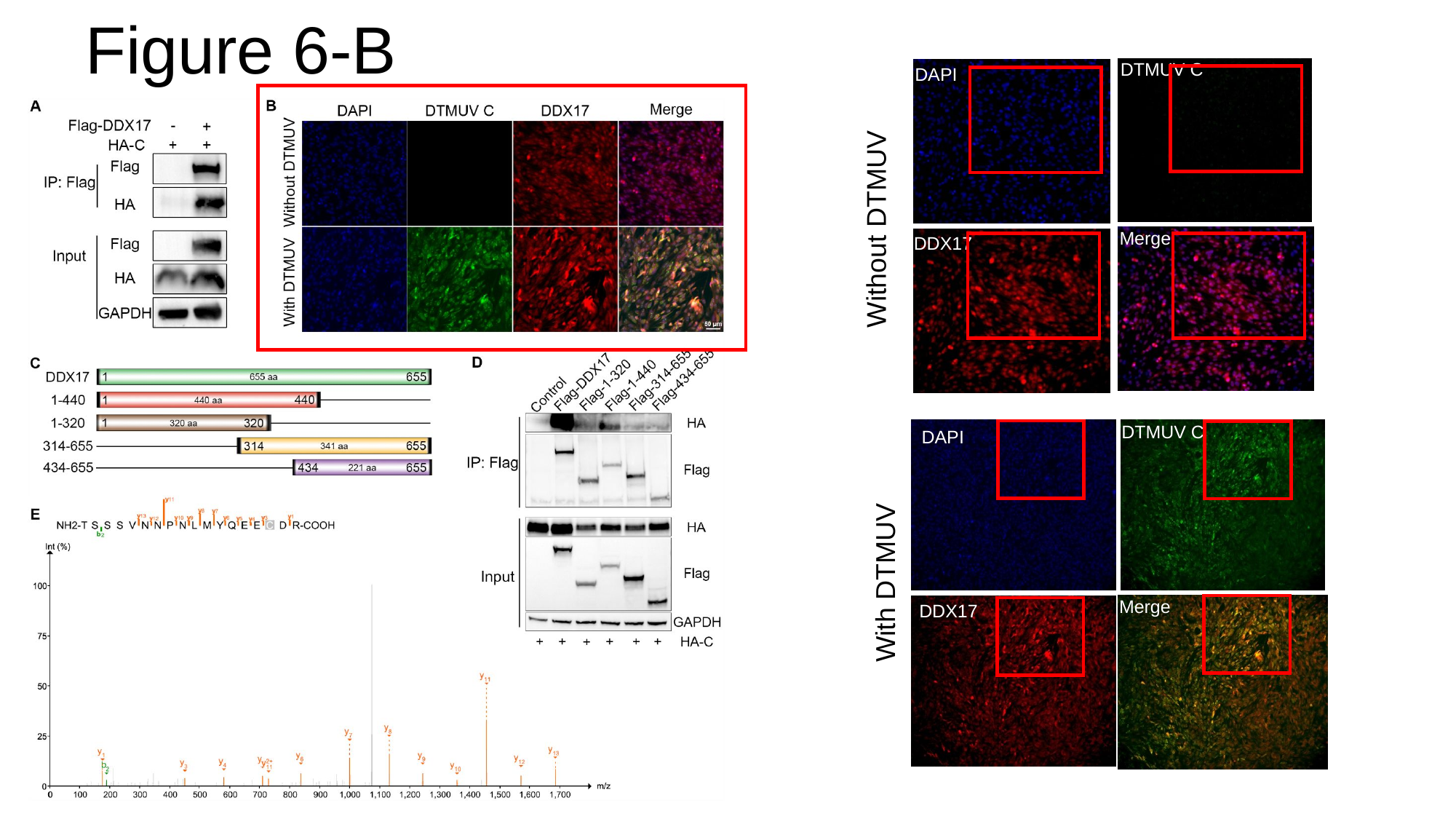

# Figure 6-B
DTMUV C
DAPI
Merge
DDX17
Without DTMUV
DTMUV C
DAPI
With DTMUV
Merge
DDX17

## Slide 8
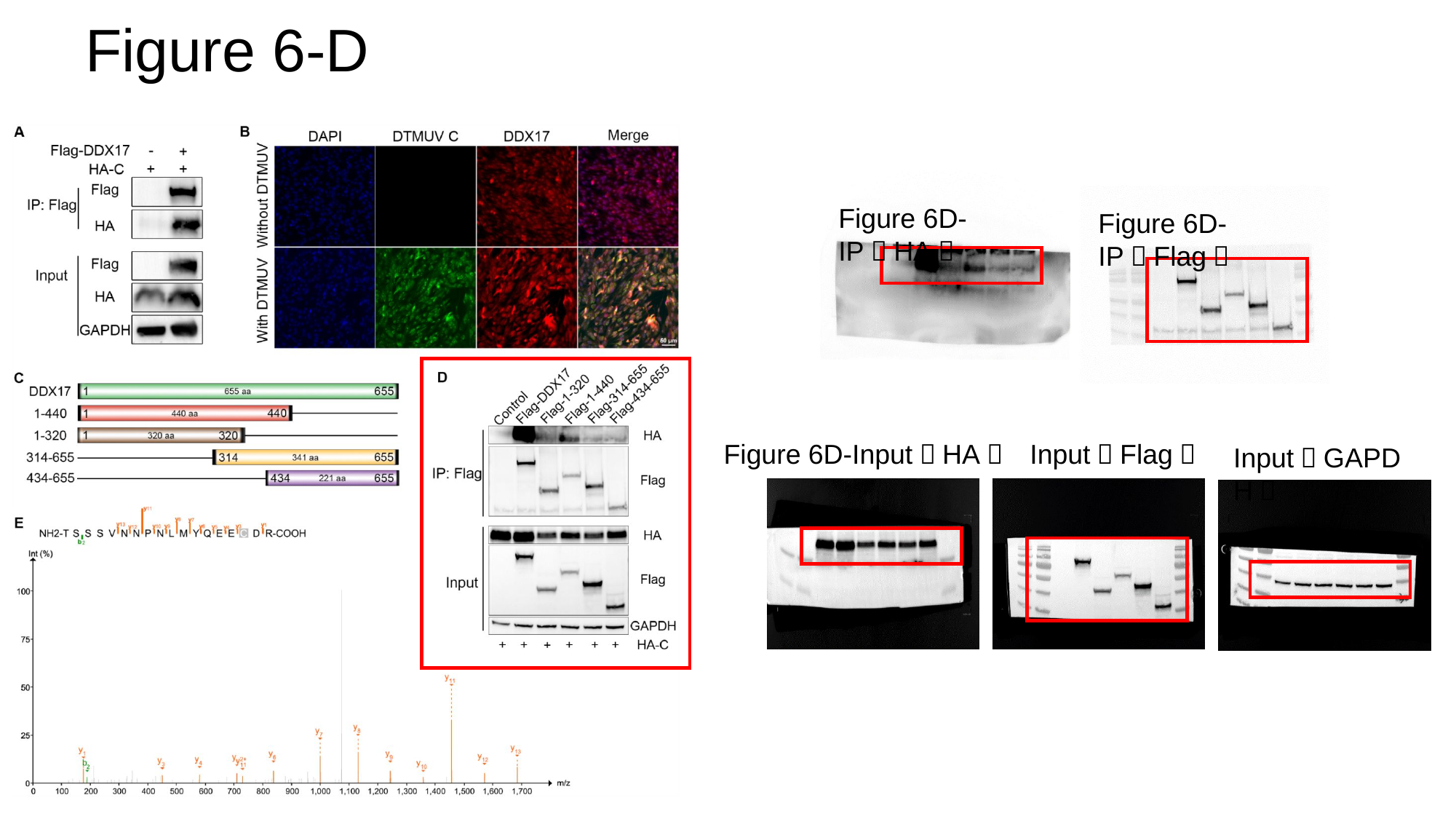

# Figure 6-D
Figure 6D-IP（HA）
Figure 6D-IP（Flag）
Figure 6D-Input（HA）
Input（Flag）
Input（GAPDH）
